# Socioeconomic and demographic factors associated with failure in *Helicobacter pylori *eradication using the standard triple therapy 

**Published:** 2021

**Authors:** Edgar Peña-Galo, Jesús Gotor, Yamal Harb, Montserrat Alonso, Javier Alcedo

**Affiliations:** 1 *Aragón Health Research Institute (IIS Aragón), Spain*; 2 *Department of Digestive Diseases, Miguel Servet University Hospital, Zaragoza, Spain*; 3 *Department of Digestive Diseases, Barbastro Hospital, Huesca. Spain*

**Keywords:** *Helicobacter pylori*, Demographic factors, Socioeconomic factors, Eradication failure, Standard triple therapy.

## Abstract

**Aim:**

To evaluate the influence of socioeconomic and demographic factors on the eradication rate of *H. pylori*, using standard triple therapy

**Background:**

the efficacy of the standard triple therapy (STT) for *H. pylori* eradication has decreased with the rise of antibiotic resistance. Other factors could influence the eradication failure, although available results are conflicting.

**Methods:**

Retrospective study, including adults with *H. pylori* infection treated de novo with STT (proton pump inhibitor, amoxicillin and clarithromycin). Eradication success was assessed by ^13^C-urea breath test. Demographic and socioeconomics variables were evaluated and correlated with eradication treatment outcome. The confounder variables were controlled by logistic regression analysis.

**Results:**

Out of 902 patients with *H. pylori* diagnosis, 693 met inclusion criteria (average age 53 years; females 55.2%). Non-significant differences were observed in relation to economics income between rural and urban areas (p=0.316). The eradication rate of *H. pylori* was 71.1%: male 78.9% vs female 65.9%, urban area 73.4% vs rural area 64.1%. With reference to age, income and nationality, the eradication rates were similar in all groups. According to logistic regression analysis, females had almost twice more likelihood of eradication failure in relation to males (OR 1.92; 95%CI: 1.38–2.72); and rural residents had OR 1.55 (95%CI: 1.03–2.33) for having eradication failure in contrast with urban population.

**Conclusion:**

Female gender and rural residence are factors associated with H. Pylori eradication failure with standard triple therapy

## Introduction

*Helicobacter pylori* (*H. pylori*) is a gram-negative bacterium able to colonize the gastric and duodenal mucosa provoking inflammation and immune response, with pH alteration. It has been related to the appearance of gastric atrophy and intestinal metaplasia, peptic ulcer disease, gastric adenocarcinoma and low-grade malignant B-cell lymphoma (1). According to worldwide estimations, *H. pylori* infection affects around 44.3% of the general population (95%CI: 40.9-47.7%), being more prevalent in developing countries (2,3). Epidemiological studies show higher infection rate in populations with elevated percentage of multi-ethnic immigration, and inversely proportional to economic level, education and the quality of potable water (4,5).

The standard triple therapy (STT) has been the regimen preferentially used to eradicate the *H. pylori* infection. It is based on the association of proton pump inhibitors (PPI) with two antibiotics: amoxicillin and clarithromycin. This combination is still successfully used in areas with low resistance to clarithromycin (<15%) (6-11). Nevertheless, there are factors which can reduce the eradication rate, the best known being the drug combination, the time of treatment, the adhesion to the therapy, and the resistance to clarithromycin in the population (12). Other conditions like demography or clinical and socio-economics features, which can result in a reduction of the treatment effectiveness, are also proposed. This group includes gender, age, ethnicity, profession, socioeconomic status, tobacco smoking, alcohol consumption, body mass index, comorbidity, previous consumption of antibiotics, and some genetic as well as geographical determinants (13-21). Even so, the results vary widely around the world.

In some studies with Asian populations (13,14,18) including more than 5200 patients treated with STT, it was concluded after a multivariant analysis that some professions (especially farmers) and female gender show less treatment success. Kim et al. (14) defined an odds ratio (OR) of 1.69 for the failure in woman and an OR of 1.61 for smokers. These results have not been confirmed in another recent study in the American population (22), where the only factor which could reduce the eradication rate was the exposition to antibiotics for the six months prior to the treatment.

Considering everything mentioned, there is a clear need for new studies to clarify which factors are associated with the failure of the treatment, or which population is at higher risk. Along these lines, we began to investigate a population with higher resistance to clarithromycin if it is possible to define socioeconomic and demographic factors that would determine differences in the eradication rate of *H. pylori*, using the STT. 

## Methods

**Design and study population**

A retrospective study in two Spanish health areas (380 718 and 109 438 inhabitants, respectively) was carried out. Adult patients (more than 18 years old) with de novo diagnosis of *H. pylori*, treated with STT and re-evaluated after treatment, were enrolled.

**Evaluation and treatment of** ***H. pylori*** **infection**

The diagnosis of *H. pylori* infection was considered with ^13^C-Urea Breath Test (UBT) positive. The STT regimen was amoxicillin 1gr/12h, clarithromycin 500 mg/12h and Omeprazole 20mg/12h, for 7 days. The eradication was evaluated by ^13^C-UBT at least 4 weeks after the conclusion of eradication treatment, no later than 12 months.

**Variables**

The demographic variables assessed were gender, age and nationality. The socioeconomic ones were income and urban or rural residence. The eradication result (value of the ^13^C-UBT post-treatment) was also determined. The demographic information was collected from the patient individual electronic clinical records. The urban/rural origin was established based on the address post-code of each patient. An urban area was considered as that with more than 10 000 inhabitants (according to Spanish Statistic Institute and Geography National Institute criteria). The economic status was estimated from the six categories ranking (001 to 006) that is shown in the Individual Spanish Health Card (ISHC). This classification corresponding with the increasing rate of pharmaceutical contribution, is in turn directly related with the level of income (001 the lowest and 006 the highest).

**Statistical analysis**

The data were analysed using the Pearson chi-squared test ( ^2^) to assess the correct distribution of the variables. In order to develop a multivariate analysis, the variables with multiple categories were dichotomized. For age, the cut point was the average age, being 18-52=0 and 53 or more=1. The yearly income was coded as 0 for 12001 euros or more and 1 for the annual income of less than 12001 euros. The nationality was defined as Spanish=0 and others=1. With reference to gender, being a woman was considered as the risk factor. These variables were related to *H. pylori *eradication using the odds ratio (OR) with 95% confidence interval (CI) to measure the association. A logistic multiple regression model was adopted to control the confounder variables. Statistical significance was set at p-value<0.05. 

**Ethics**

The data of the study were strictly confidential and only the researchers had access to them, according to the Spanish personal data protection law. The study was carried out in accordance with the principles of the 1975 Declaration of Helsinki (6th revision, 2008). The protocol study was approved by the Regional Ethics Committee (CEIC Aragon: C.P. - C.I. PI18/360).

## Results

**Figure 1:**
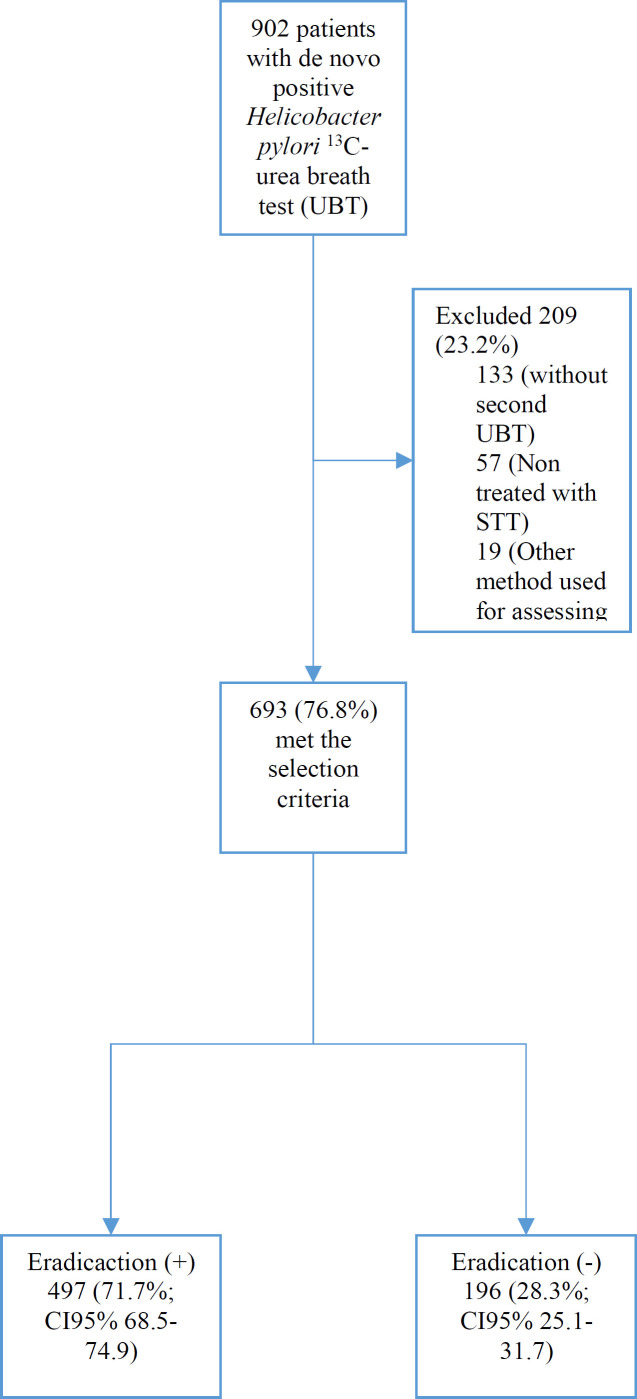
Patients’ Flowchart

A total of 902 patients had a positive ^13^C-UBT, and 209 were excluded (23.2%) for the following reasons: 133 did not have a second UBT, 57 were treated with a regimen different from STT, and 19 had a different method of evaluating eradication to ^13^C-UBT (Figure 1).

Six hundred and ninety-nine patients were finally enrolled. The average age was 53 years old (range 18-83); 55.2% were women and 44.8% men. After stratifying the gender by age groups and income, no statistical differences were observed (*p*=0.814 and *p*=0.646, respectively). However, we found differences in the gender distribution according to nationality (*p*=0.028), with the male gender being more frequent among the cases from Africa, Asia and Eastern Europe and the female among Latin American patients. The main nationality was Spanish, representing 82.7% (men 83.4% and women 82.1%). Regarding urban/rural distribution, our results shown differences among age groups and nationalities (*p*=0.026 and *p*<0.001, respectively). In the urban area, the Latin American immigrant population was more frequent, while in the rural area it was the African origin. With regards to income, no differences were observed between urban and rural population (*p*=0.440) (Table 1).

The global eradication rate of *H. pylori* in our patients was 71.1% (95%IC 68.5-74.9), being higher in men [78.9% (95%IC 74.0-83.1)] than in women [65.9% (95%IC 61.1-70.5)]. After comparing the urban/rural origin, we observed that the urban area had an eradication rate of 73.4% (95%IC 69.7-76.9) being almost 10% higher than that achieved in rural area, 64.1% (95%IC 55.5-71.8). In relation to age, income and nationality, the percentage of eradication was similar among the study groups (Table 2). The logistic regression analysis concluded that women were almost twice as likely for the eradication failure in comparison with men (OR 1.92; 95%IC 1.38-2.72). Additionally, the rural population showed 55% more eradication failure in contrast with urban population (OR 1.55; 95%IC 1.03-2.33). In our study, the variables of age, income and nationality were not associated with therapeutic failure (Table 2).

## Discussion

In the present study, results were obtained in favour of the existence of socioeconomic determinants associated to *H. pylori* eradication failure using STT. In the geographical area where the study was carried out, other authors have noticed a clarithromycin resistance of higher than 15%^8^. This may justify the 71% of therapeutic efficacy in the analysed sample, lower than the standards considered acceptable to an eradicator regimen for the bacteria. In addition, after evaluating other possible factors involved, we observed that upon logistic regression analysis, including factors of age, gender, income, nationality and area of living, being a woman and living in a rural area could decrease the success of *H pylori* eradication in an independent way.

**Table 1:** Distribution by gender and living place urban/rural of the study variables

Variables	Total	Male	Female	p-value^‡^	Urban	Rural	p-value^ ‡^
	n (%)	n (%)	n (%)		n (%)	n (%)	
Stratified age				0.814			
18 – 25 26 – 35 36 – 45 46 – 55 56 – 65 ≥ 66	23 (3.3) 82 (11.8) 136 (19.6) 151 (21.8) 131 (18.9) 170 (24.5)	12 (3.9) 37 (12.0) 64 (20.8) 61 (19.8) 56 (18.2) 78 (25.3)	11 (2.9) 45 (11.7) 72 (18.7) 90 (23.4) 75 (19.5) 92 (23.9)		16 (2.8) 66 (11.7) 102 (18.1) 122 (21.6) 118 (20.9) 141 (25.0)	7 (5.5) 16 (12.5) 34 (26.6) 29 (22.7) 13 (10.2) 29 (22.7)	0.026*
ISHC^†^				0.646			
001 002 003 004 005 006	26 (3.8) 275 (39.7) 272 (39.2) 112 (16.2) 2 (0.3) 6 (0.9)	14 (4.5) 121 (39.3) 118 (38.3) 53 (17.2) 0 (0.0) 2 (0.6)	12 (3.1) 154 (40.0) 154 (40.0) 59 (15.3) 2 (0.5) 4 (0.6)		18 (3.2) 226 (40.0) 218 (38.6) 96 (17.0) 2 (0.4) 5 (0.9)	8 (6.2) 49 (38.3) 54 (42.2) 16 (12.5) 0 (0.0) 1 (0.8)	0.440
				0.028*			
Nationality African Asian Easthern European Latin American Spanish	24 (3.5) 5 (0.7) 28 (4.0) 63 (9.1) 573 (82.7)	13 (4.2) 3 (1.0) 17 (5.5) 18 (5.8) 257 (83.4)	11 (2.9) 2 (0.5) 11 (2.9) 45 (11.7) 316 (82.1)		15 (2.7) 5 (0.9) 21 (3.7) 63 (11.2) 461 (81.6)	9 (7.0) 0 (0.0) 7 (5.5) 0 (0.0) 112 (87.5)	<0.001*
Total	693 (100.0)	308 (100.0)	385 (100.0)		565 (100.0)	128 (100.0)	

^‡^Chi square. ^†^ISHC: Individual Spanish Health Care (code of pharmaceutical, contribution directly related with the the level of income). *p<0.05

**Table 2:** Univariate analysis and logistic regression to determine the association between socio-demographic variables and* H pylori *eradication failure with STT (n=693)

Variables	Positive Eradication		Crude OR	CI95%	Adjusted OR^†^	CI95%
	n (%)	CI95%				
Gender Male Female	243 (78.9) 254 (65.9)	(74.0-83.1) (61.1-70.5)	Ref 1.92	1.38 – 2.72	1.98	1.39 – 2.80
Age 18 – 52 years ≥ 53 years	252 (70.9) 245 (72.5)	(66.1-75.5) (67.5-76.9)	Ref 0.92	0.66 – 1.29	0.84	0.54 – 1.33
Income level Mid-High* Low-Very low**	282 (71.9) 215 (71.4)	(67.3-76.2) (66,1-76,2)	Ref 1.02	0.73 – 1.43	1.13	0.73 – 1.76
Nationality Spanish Other	410 (71.5) 87 (72.5)	(67.7-75.1) (63.9-79.7)	Ref 0.95	0.61 – 1.48	0.93	0.58 – 1.50
Living place Urban Rural	415 (73.4) 82 (64.1)	(69.7-76.9) (55.5-71.8)	Ref 1.55	1.03 – 2.33	1.59	1.05 – 2.41

^†^Logistic regression analysis. Ref: Reference value. * ISHC codes 001 and 003 (corresponding to non-contributory pensions and yearly income lower than 18.000€). **Other ISHC codes (corresponding to contributory pensions, civil servants and yearly income higher than 18.000€)

The female gender in our study showed a risk of therapeutic failure almost twice the rate of male gender (OR 1.98; 95%CI 1.39-2.80). This is consistent with results of a systematic world revision which concludes that the average resistance rate to clarithromycin was 20.5% in women versus 15.5% in men, the difference being statistically significant (*p*<0.001; OR 1.4, 95%IC 1.2-1.5) (23). A study developed in the Spanish southern population also observed an association between female gender and *H. pylori* clarithromycin resistance (OR 1.71; 95%IC 1.007-2.913) (15). In the same vein, several recent studies performed in Asian population have found that the risk of eradication failure is lower among females, with OR 1.69 to 1.73 (18-20). Conversely, some authors evaluating populations with lower rate of clarithromycin resistance could not conclude that gender was a risk factor involved in the resistance, or influences the eradication therapy success, when STT regimen is used (22). 

The results are difficult to explain in this area of research. In Navarro-Jarobo et al. (15) study, genetic mutations associated with clarithromycin resistance (essentially A2143G in 82.3% and A214G in 7.1% of the cases) were detected in a related population, but the authors could not define the gender distribution of these mutations. In a Korean population, Hwang et al. (24) had previously revealed that mutation A143G was related to *H. pylori* eradication failure in 87.5% of patients treated with triple therapies containing clarithromycin. Additionally, this gene was mainly expressed among females. Recognising potential genetics factors, other studies found differences in the biologic behaviour of genetic metabolizer of CYP2C19 regarding gender, but it could not be unequivocally linked with the result of the eradication (17,19).

Examining other potential causes, it is proposed that the more frequent use of antibiotics among women, essentially to solve urinary or gynaecologic infections, could justify the results. In fact, the previous use of macrolides, especially during time periods longer than two weeks, is associated with eradication failure.^18^ Furthermore, a study in a Northern Canadian population (25) showed worse compliance of treatment among women compared with men, although no multivariate analysis was performed to exclude bias. The adherence to the eradication therapy is an essential factor for its success (26). However, it cannot be assessed with accuracy in our study due to its retrospective design, hence unduly affecting the results.

After analysing the role of the variable of place of living, we have obtained a rise in the eradication failure in rural population as contrasted to urban inhabitants (OR 1.59; 95%IC 1.05-2.41). Nevertheless, the variable acts heterogeneously in the limited publications that research it. While in China, Cai et al.^13^ found that farmers compared to civil servants had ten times the likelihood of failure in the eradication treatment with STT, in Korea, Kim et al. (14) did not find differences in the function of the living place variable. However, there is some additional information published which could be related to poorer therapeutic performance in rural areas. The transmission of the bacteria in urban area seems to occur basically from person to person, while in rural populations, water and contaminated food are added as likely transmission routes (27). Perhaps for similar reasons, the infection and reinfection are more frequent too (28,296). According to our findings, factors like age distribution and income could not explain the differences. 

In this study, we evaluated the effectiveness of the eradication treatment with STT in both populations, rural and urban, analysing the influence of the socio-demographic factors rarely addressed so far, especially in a non-Asian population. As has been mentioned above, the retrospective design does not allow to provide a random sampling of the population in order to reduce the potential bias. On the other hand, although the antimicrobial resistance in our population is presumably high, according to the information in nearby geographical areas, this topic was not assessed in the patients enrolled in the study before the eradication treatment.

In a geographical area where *H. pylori* eradication rate with STT is lower than 80%, the female gender and living in a rural area are potential individual risk factors associated with therapeutic failure. This could have repercussions in the antibiotic pattern choice in certain groups of patients. Further prospective studies are needed to ensure if the same factors could affect the effectiveness of other therapeutic regimens.

## Conflict of interests

The authors declare that they have no conflict of interest.
